# Genetic Diversity and Dye-Decolorizing Spectrum of *Schizophyllum commune* Population

**DOI:** 10.4014/jmb.2006.06049

**Published:** 2020-08-14

**Authors:** Yongjun Choi, Ha Thi Kim Nguyen, Tae Soo Lee, Jae Kwang Kim, Jaehyuk Choi

**Affiliations:** Division of Life Sciences, College of Life Sciences and Bioengineering, Incheon National University, Incheon 22012, Republic of Korea

**Keywords:** *Schizophyllum commune*, dye decolorization, population genetics, biodiversity

## Abstract

Synthetic dyes are widely used in various industries and their wastage causes severe environmental problems while being hazardous to human health, leading to the need for eco-friendly degradation techniques. The split-gill fungus *Schizophyllum commune*, which is found worldwide, has the potential to degrade all components of the lignocellulosic biomass and is a candidate for the treatment of synthetic dyes. A systematic molecular analysis of 75 Korean and 6 foreign *S. commune* strains has revealed the high genetic diversity of this population and its important contribution to the total diversity of *S. commune*. We examined the dye decolorization ability of this population and revealed 5 excellent strains that strongly decolorized 3 dyes: Crystal Violet, Congo Red and Methylene Blue. Finally, comparison of dye decolorization ability and the phylogenetic identification of these strains generalized their genetic and physiological diversity. This study provides an initial resource for physiological and genetic research projects as well as the bioremediation of textile dyes.

## Introduction

Synthetic materials such as dyes, polymers and chlorinated hydrocarbons have been extensively used in many modern industries and their wastes are causing severe environmental pollution while being harmful to human health [1, 2]. There is a pressing need to develop eco-friendly treatment techniques to degrade these wastes. Biological treatments serve as a cost-effective and eco-friendly solution for this pollution [1, 3]. White-rot fungi (WRF) are attractive for this purpose because of their capacity to degrade such materials by virtue of their extracellular enzymes [3-5]. WRF are natural decomposers of all components of wood, including the recalcitrant, aromatic, and heterogeneous lignin polymers. Especially, lignin modification is possible because of a wide array of extracellular oxidoreductases produced by WRF [6, 7].

Among these WRF is the split-gill fungus *Schizophyllum commune*, which is considered as a rising candidate for bioremediation by dye degradation [8]. *S. commune* is a mushroom- forming fungus for which the whole genome sequence was published in 2010 [9]. This fungus can be easily cultivated in the laboratory; its life cycle completes on synthetic media within 10 days, and it offers a variety of molecular tools. Moreover, *S. commune* has been used as a model organism of fruiting body formation in mushroom [8, 9], and it is distributed around the world [9]. Like other WRFs, *S. commune* might strongly express the lignocellulose degrading ability. Since its genome contains 240 gene candidates for glycoside hydrolases, 75 for glycosyl transferases, 16 for polysaccharide lyases, 17 for expansin-related proteins, 30 for carbohydrate esterases, and 16 for lignin-degrading oxidoreductases [9], *S. commune* has the genomic potential to degrade all components of the lignocellulosic biomass. At least 150 genera of woody plants are substrates for *S. commune*, but it also colonizes softwood and grass silage [9].

*S. commune* has successfully established itself around the world, indicating its wide range of genetic diversity. The genetic diversity of 195 *S. commune* strains has been investigated using the internal transcribed spacer (ITS) and intergenic spacer (IGS) regions of the rDNA sequence, revealing three major geographic groupings within the global population: North/Central America (NAM), South America (SAM), and Europe/Asia/Australia (EAS)[10]. In the East Asia area, analyzing 10 Korean and two Chinese *S. commune* strains showed that most of the Korean and Chinese strains formed a different clade from the control group (i.e., South American strains used in the study of James *et al*., 2001) [10, 11]. This high genetic diversity might be explained by the high mutation rate in the genomes of the *S. commune* populations from America and Russia [12].

With such diversity and widespread distribution, *S. commune* serves as a good resource for genetic research as well as for expanding the range of possibilities for biotechnological products including those in the dye degradation industry. However, while there are many studies focusing on using WRF for dye degradation [4, 5], very little research has been conducted on *S. commune*. Few *S. commune* strains are known to have the ability for dye decolorization and dye degradation, such as IBL-06, F17 [13-18]. Therefore, study of the dye degradation abilities of *S. commune* species is a promising area with great potential for use in industrial processes such as the biodegradation of complex substances and pollutants, and the processing of agricultural products and byproducts of biofuel production.

In this study, we extracted ITS sequences from 81 *S. commune* strains and investigated their genetic diversity in comparison with the global population using phylogenetic tree and haplotype network analysis. Moreover, we evaluated their decolorizing ability for different textile dyes to show their physiological diversity, and selected the strains best fitting this criteria for future use as a major research resource and to improve understanding of the mechanism of oxidase secretion in eukaryotes.

## Materials and Methods

### Fungal Strains and Culture Media

All 81 strains of *S. commune* in this study were obtained from the Culture Collection of Mushrooms (CCM) at Incheon National University and are shown in Table S1. The medium used for strain culture is potato dextrose agar (PDA, 39 g in 1,000 ml distilled water; BD Difco). The medium was sterilized under high-pressure condition at 121°C for 15 min. *S. commune* strains were cultured on this medium at 25°C for 5~7 days in dark condition, and stored at 4°C until being used for experiment.

### Fungal DNA Extraction

To extract genomic DNA from *S. commune*, strains were grown on semipermeable cellophane membrane-covered plates [19]. *S. commune* minimal medium (SMM, glucose 20 g, KH_2_PO_4_ 0.46 g, K_2_HPO_4_ 1 g, MgSO_4_•7H_2_O 0.5 g, Trace element solution 1 ml, FeCl_3_ solution 1ml, L-Asparagine 1.5 g, filter-sterilized thiamine 1.2 ml, agar 20 g) was autoclaved then aliquoted into 9 cm petri dishes. The 6 x 6 cm cellophane membranes were placed on the surface of media-containing plates. The inoculum, which had been stored at 4°C, was cut into 4 pieces (5 mm each) and placed on new media-containing cellophane membranes, then incubated at 25°C within 5 days in dark condition. Using a scalpel, only cultured mycelium was collected from the cellophane membranes, then pulverized in liquid nitrogen using a mortar and pestle. The pulverized mycelium powder was transferred to a 1.5 ml Eppendorf tube and genomic DNA was extracted following fungi protocol using a Macherey-Nagel NucleoSpin Plant II Kit. Twenty mg of mycelium powder was treated with cell lysis solution for 30 ~ 60 min. Then, 2.5 units of RNase and 0.03 unit of proteinase K were added and incubated for 30 min to increase the purity of the DNA.

### Sequence Analysis 

ITSs in eukaryotes are two nucleotide regions existing between the 18S, 5.8S and 26S ribosomal RNA encoding sequence, together forming the rRNA cistron which is transcribed as a unit by RNA polymerase I. The rRNA sequences can be split and the ITS removed in the post-transcriptional step. However, for sequence analysis in phylogenetics, the sequence of ITS used consists of both 2 ITS regions and the 5.8S rRNA encoding sequence. The ITS sequence has been found to be a useful genetic marker for distinguishing fungal species (Smith *et al*., 2010).

To amplify the ITS region with DNA product size around 500-800 bps, 50 ng of gDNA template of obtained mushroom and 2 primers ITS1 (5’-TCCGTAGGTGAACCTGCGG-3’) and ITS4 (5’-CCTCCGCTTATTGATATGC-3’) were used. The reaction occurred in the presence of 1 unit of Phire Hot Start II DNA Polymerase (Thermo Scientific). PCR reaction was performed with 3 steps: initial denaturation at 98°C, 30 sec, 1 cycle; denaturation at 98°C, 5 sec; annealing at 53~55°C, 5 sec; extension speed of 10-15 s/kb, 35 cycles; final extension at 72°C, 1 min, 1 cycle; holding sample at 4°C.

PCR products were purified according to the protocol of AccuPrep’s PCR/Gel DNA Purification Kit. The precise DNA sequence was confirmed in both directions of ITS based on standard sequencing by Macrogen Co. One final sequence was obtained based on the peak quality obtained by electrophoresis of the bidirectional sequence.

### Phylogenetic Analysis of Korean Domestic and Foreign White-Rot Fungi

In addition to the 81 ITS sequences obtained from the CCM of Incheon National University, 137 ITS sequences of *S. commune* published in the NCBI were also included for systematic analysis. The list of *S. commune* strains in NCBI is shown in Table S2. Multiple sequence alignment analysis was performed using a Muscle sequence aligner (ver. 3.8.31). The aligned sequences were trimmed by using the trimAL program (ver. 1.4. rev15) on the Linux server (Ubuntu 16.04.5 LTS). At this time, the gap threshold option (-gt) was set as 0.75, which removes all positions in the alignment with gaps in 25% (1-0.75) or more of the sequence. RAxML (ver. 8.2.12) was used to draw the maximum-likelihood tree. The options for the CAT model of rate heterogeneity (‘-m GTRCAT’) and for automatic bootstrapping (‘-N autoMRE’) were used to select the most suitable analytical model. For visualization, the ape and ggtree packages in R were used. To analyze the nucleotide and haplotype diversity, the pegas package in R was used.

### Analysis of Synthetic Dye Decolorization Ability of *S. commune* Strains 

Four types of dye used in the screening test were classified into 4 chromophore types: diazo-based Congo Red, anthraquinone-based Remazol Brilliant Blue R, thiazine-based Methylene Blue, and triphenylmethane-based Crystal Violet.

Fungal culture medium (PDA) was supplemented with 20 mg/l dye, sterilized, then aliquoted into 6 cm petri dishes to be ready for screening test. A single 4.4 cm diameter cork borer was used to inoculate each strain on each dye-containing medium with the same size of inoculum. The inoculation was repeated twice, placed in a sealed box to maintain humidity, and cultured at 27°C in dark condition. Samples were observed every day and the bottom surface of the medium was scanned every 3 days for decolorization ability and activity comparison. The inoculum dishes were compared with original dye-containing media, and the level of dye-decolorization was determined as (+), for ‘has ability to decolorize,’ or (-) for ‘has no ability.’ In addition, high decolorization ability was recorded as around 6 cm, and low, around 3 cm.

Liquid medium (Potato Dextrose Broth) was prepared similar to solid media without agar. Each 10 ml of dye-containing liquid media was transferred to a 50 ml conical tube. Then, the same amount of inoculum was inoculated into each medium using a 4.4 mm diameter cork borer. The inoculated tubes were incubated at 27°C and 150 rpm in dark condition. The fungal cultures containing dyes were filtered through Whatman filter papers 42 (cat. no. 1442-090) at 12 days post inoculation, then the culture was secured. The filtered culture was transferred to a 1 mL cuvette and the absorbance was measured by using a NanoDrop 2000c instrument. The absorbance wavelengths for each dye, Congo Red, Remazol Brilliant Blue R, Methylene Blue and Crystal Violet, were 485, 595, 620, and 560 nm, respectively. The decolorization rate was calculated based on the absorbance in the control dye-containing medium without strains and the absorbance in medium in which strains were cultured. To standardize the degree of bleaching, various concentrations of control dye media were prepared to measure the absorbance.

## Results

### Species Identification of White-Rot Fungal Strains Isolated from CCM Bank

The genomic DNA of 81 *S. commune* strains obtained from CCM bank were extracted and their ITS sequences were determined. The BLAST search confirmed that all of the 81 sequences had more than 98% identity and high similarity with the ITS sequence of *S. commune*.

**Fig. 1:**
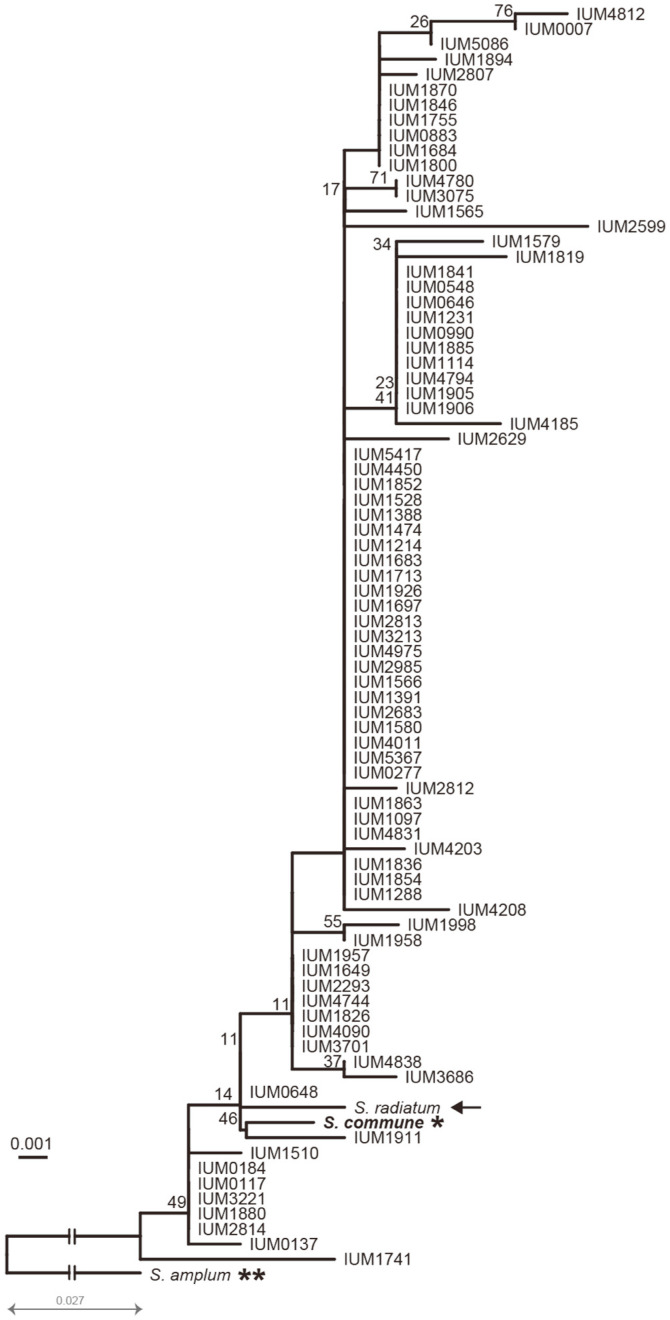
The phylogeneic analysis of *S. commune* population. A total of 81 strains from the CCM bank were used. *S. commune* H4-8 strain (*) and two outgroup strains, *S. amplum* (**) and *S. radiatum* (black arrow), were added. The broken lines indicate the shortening of the *S. amplum* branch. Maximum likelihood tree was generated by RAxML using the CAT model and automatic bootstrapping options.

In order to determine the genetic relation among these strains, four other closely related species in *Schizophyllum* were included as an outgroup: *S. fasciatum* (Acccession No: AY179593.1), *S. umbrinum* (acccession no: AY179594.1), *S. amplum* (Acccession No: DQ097353.1) and *S. radiatum* (Acccession No: LN714603.1). *S. commune* (H4-8) was used as a standard strain. A total of 86 nucleotide sequences were used for multiple sequence alignment. The maximum likelihood-based phylogenetic tree was drawn to examine the genetic relation between 81 white-rot fungus strains. In 86 nucleotide sequences tested, two mushroom species, *S. facsciatum* and *S. umbrinum*, were very far from the 82 other strains. As a result of the pairwise distance calculation based on the branch length, *S. umbrinum* was 0.193 away from *S. facsciatum*, but was 0.766 away from the rest of 82 *S. commune* strains on average. Therefore, these two outgroup strains were removed and the phylogenetic analysis was performed again using only the 84 remaining strains (Fig. 1). The *S. commune* H4-8 was the farthest from the outgroup species *S. amplum* (0.065) and very close to the average distance of other strains (0.012 ± 0.004, *n* = 82). This indicates that the 81 strains obtained from CCM genetically belonged to *S. commune* species. Interestingly, *S. radium*, which was known as a pathogen infecting humans, was systematically bound together with H4-8 strain, suggesting that under ITS analysis, *S. radium* is the same species with *S. commune*.

### Genetic Diversity of *S. commune* Population

To investigate the genetic diversity of *S. commune* strains isolated in Korea, six ITS sequences of the strains isolated from overseas were excluded from 81 nucleotide sequences. A haplotype network was then constructed for the remaining 75 domestic strains (Fig. S1). There were 44 haplotypes with a diversity score of 0.92505 (Table 1). To reveal the correlation between haplotype and regional distribution, a pie chart was created for each region (Fig. S1). However, there was no specific correlation between haplotypes and regional distribution of domestic strains because most of the haplotypes seemed distributed evenly.

To compare the genetic diversity of domestic and foreign *S. commune* strains, the ITS sequences of 135 strains at NCBI, which has different location information, were collected (Table S2), and then divided into 6 regions at the continental level (Table S3). Of the 135 sequences collected, 4 nucleotide sequences from Korea were excluded, while the sequences of 6 foreign strains from the CCM bank and one sequence of the H4-8 strain were included. These 138 sequences in total were then fed into multiple sequence alignments. The nucleotide diversity analysis of these sequences showed a value of 0.002658, which was higher than the domestic value (0.000893, Table 1) [20]. Haplotype diversity analysis revealed 73 haplotypes and a diversity number of 0.92066, similar to that of the domestic group (0.92505) (Table 1). In comparison with the domestic group, although the number of haplotypes of the foreign group was almost doubled, the haplotype diversity was similar.

Moreover, the haplotype network of 138 foreign strains was constructed and the correlation between haplotype and its distribution was examined (Fig. S2). The largest haplotype (LV) included strains from all regions, but the major strains in this haplotype were from Europe (23/35 strains). South Asian strains composed the main part (14 strains/total 14 strains) in the 2^nd^ largest haplotype (XXVIII, *n* = 14), and became the biggest contributor (6 strains/total 11 strains) in the 3^rd^ largest haplotype (XLII, *n* = 11). However, the European and South Asian strains also existed in other small haplotypes. Therefore, specificity was found between regional distribution and haplotypes from some areas of South Asia and Europe.

**Fig. 2:**
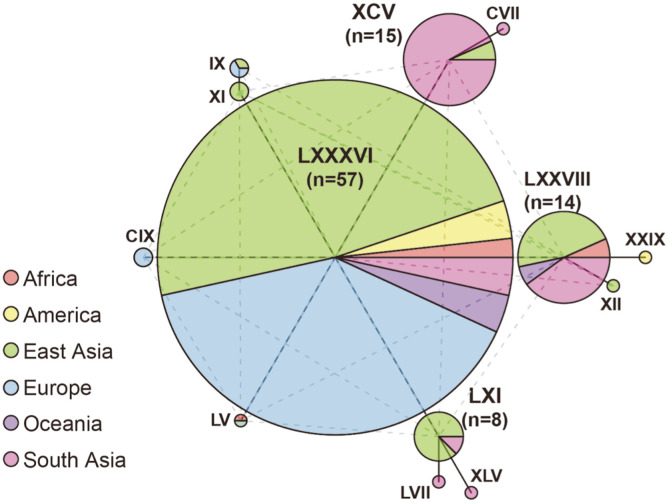
Haplotype network analysis of *S. commune* strains. A total of 217 *S. commune* strains including domestic and foreign groups were analyzed. Total haplotype number is 113 but only the haplotypes containing more than two strains are displayed.

To determine how the diversity of the Korean *S. commune* population affects the genetic diversity of *S. commune* around the world, the entire 217 strains (including 75 domestic and 138 foreign groups and four Korean sequences obtained from NCBI) were extended and analyzed for diversity (Table 1 and Fig. 2). The fact that the number of haplotypes of total strains (113) was almost equal to the sum of the domestic haplotypes (44) and foreign haplotypes (73) suggested that there might be very few overlapped haplotypes between the domestic and foreign groups when they were combined into a single group. Indeed, among 113 haplotypes among the total strains, there were only five haplotypes comprising both domestic and foreign strains; meanwhile, 39 haplotypes of total group were composed completely of domestic strains and 69 haplotypes of total group were from foreign group. Five overlapped haplotypes in total group were LXXXVI, XCV, LXXVIII, LXI, and IX (Fig. 2). The LXXXVI haplotype was the largest haplotype in total group with 58 strains of which 20 strains from domestic population and 35 strains from foreign group (Fig. 2). Moreover, the nucleotide diversity of all strains was 0.00283, just slightly higher than that of the foreign population 0.00266 (Table 1) while the total number of haplotypes (113) was higher than that of the foreign strains (73). Taken together, it suggested that the domestic *S. commune* strains had unique DNA sequences, and showed relatively low contribution in the nucleotide diversity but high contribution to the haplotype diversity of total group. In addition, the South Asia-centric haplotypes (XXVIII and XLII in foreign group) appearing as important ones in the foreign population still maintained uniqueness since these haplotypes’ members constitute a major part of XCV (14/15 strains) and lesser part of LXXVIII (5/15 strains) haplotypes in total group. The combination of domestic and foreign groups formed one new overlapped haplotype, LXI, which was composed mainly of Korean strains (7/8 strains from Korea, 1/8 strain from South Asia), and made the East Asia region a major contributor for LXXVIII haplotype (7 strains/total 15 strains were from East Asia). Thus, certain haplotypes were only displayed in the relevant separation area.

**Fig. 3:**
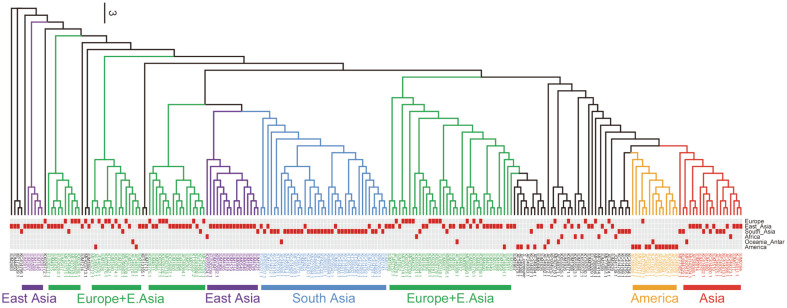
Correlation between the maximum likelihood tree and isolated regions in the world population of *S. commune*. Selected residues of ITS sequence were 501 ~ 505 bp. Maximum likelihood tree was generated by RAxML using the CAT model and automatic bootstrapping options.

To investigate the relation between regional distribution and genetic composition of total *S. commune* strains, we compared the phylogenetic tree of the total 217 strains and their regional distribution. The phylogenetic tree was drawn based on the ITS sequences of all 217 *S. commune* strains (Fig. 3). Regional distribution of each strain was displayed as a heat map and compared with the phylogenetic tree (Fig. 3). The separated area information is divided into six regions at the continental level: Europe, East Asia, South Asia, Africa, America and Oceania/Antarctica. Based on the original regional distribution of major strains, which normally occupied more than 75% of the population in each group, 7 groups could be found and named as Asian, American, European + East Asian and South Asian. For example, the South Asia group with a total of 38 strains was composed of 30 strains from South Asia (78.9%), 7 strains from East Asia and 1 strain from Oceania/Antarctica. No single clade was created for any specific region. Some strains were isolated from different regions but bound to the same clade. However, Fig. 3 shows that many strains isolated from the same region were bound to a unique clade. For example, although some South Asian strains were located far from each other on the tree, 60% of this population (30 strains/50 strains total) was classified into one clade marked with blue and named as the South Asia clade. Similar, there are clearly four clades named as East Asia (purple - 2 clades), America (orange – 1 clade) and Asia (red – 1 clade). These data suggest that the strains isolated from the same region and bound to a unique clade have high similarity in ITS sequence, or share unique sequences with each other. It means that their ITS sequences are unique by region. Besides the unique characterization of the American group (forming an individual clade – orange), two green groups, containing mainly both East Asian and European strains, indicate that strains in these regions are genetically similar. Indeed, haplotype analysis revealed that 85% (40/47 strains) of the green group between two East Asia clades belonged to haplotype LXXXVI - the largest one in the haplotype network shown in Fig. 2.

### Diverse Dye-Decolorization Ability of *S. commune* Population

Several studies have found that some *S. commune* strains have dye-decolorization and dye-degradation abilities [13-17]. Therefore, we attempted to investigate that ability in 81 new *S. commune* strains obtained from CCM. In this screening, 4 textile dyes, Congo Red, Remazol Brilliant Blue R, Methylene Blue and Crystal Violet, representing 4 chromophore types respectively, Diazo, Anthraquinone, Thiazine and Triphenylmethane, were separately incubated with each fungal strain on PDA media. Mycelium of isolated *S. commune* strains from CCM grew to the edge of a 6 cm plate within 12-15 days, around 5 days more slowly than mycelia growing on media without dye.

**Fig. 4:**
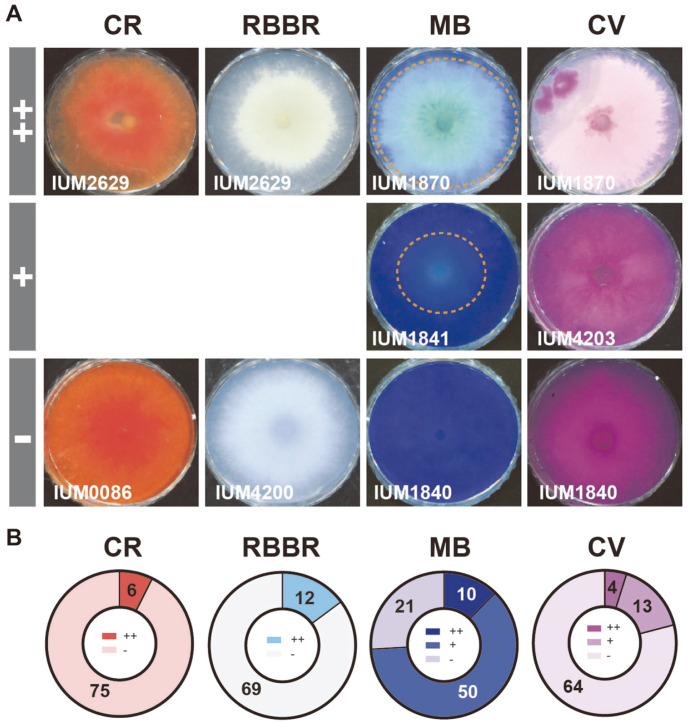
The diverse pattern of dye-decolorization of *S. commune* strains. (**A**) Selection standards of dye decolorization ability. -: no decolorization ability, +: has decolorization ability, ++: has strong decolorization ability. Decolorization was determined at 9 dpi. CR: Congo Red, RBBR : Remazol Brilliant Blue R, MB: Methylene Blue, CV: Crystal Violet. (**B**) Distribution on pie charts of the dye decolorization ability. Different intensity of colors indicates levels of decolorization. Number indicates the number of strains for the specific level.

As mycelia grew on each plate, the degree of dye-decolorization was visually confirmed and divided into three stages (Fig. 4A): ++ strong decolorization, the dye color almost disappeared from media; + has decolorization, dye color faded from media; - no decolorization, dye color remained intact on media. In case of Congo Red and Remazol Brilliant Blue R, the distinction was not clear, so it was divided into only two stages (++ and -). The strains that have the same level of decolorization ability for each dye have been counted and presented in distribution charts (Fig. 4B, when grown on Methylene Blue-containing media, in total 81 strains tested, 60 strains (74%) exhibited decolorization ability (++ and +), implying that MB contained the easiest chromophore type to be decolorized by these *S. commune* strains. In contrast, Congo Red is the most difficult one to be decolorized since only 6 strains (7.4%) showed decolorization ability among 81 strains tested. For Crystal Violet and Remazol Brilliant Blue R, 17 and 12 strains showed decolorization ability. The dye-decolorization spectrum of CCM *S. commune* population is presented in Fig. 6. In summary, nearly 75% of tested strains exhibited decolorization ability for at least one type of dye.

**Fig. 5:**
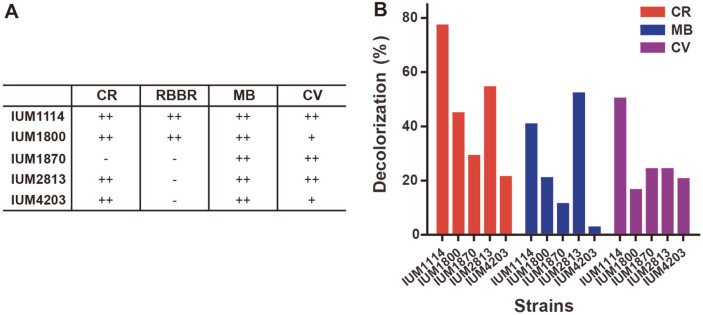
Decolorization of the selected *S. commune* strains. (**A**) List of five excellent strains that showed strong decolorization ability on dye-containing solid media. (**B**) The decolorization rate of the five strains on dye-containing liquid media including Congo Red (CR), Methylene Blue (MB), and Crystal Violet (CV).

In addition, the above decolorization results needed to be confirmed by quantitative method which can be performed using liquid culture media. To do that, five strains showing excellent dye decolorization abilities (decolorized at least 2 types of dye) were selected as listed in Fig. 5A. These 4 domestic strains, IUM 1114, IUM 1800, IUM 1870, IUM 2813, and one foreign strain, IUM 4203 from Italy, were grown on liquid media containing different dyes. The light absorbance of media containing dye before and after fungus inoculation was measured and the decolorization ratio was calculated. As shown in Fig. 5B, the dye-decolorization of 5 selected strains on Congo Red, Methylene Blue, Crystal Violet has been confirmed in liquid media. The absence of Remazol dye in this test is explained below. Among them, strain IUM1114 which showed strong decolorization of 4 dyes on solid media, was again confirmed to be an excellent strain in decolorization ability on liquid media with ability to decolorize Congo Red (77.6%), Methylene Blue (41.1%), and Crystal Violet (50.6%). A similar result was seen in case of IUM 2813, which showed decolorization values of 54.8% in Congo Red, 52.5% in Methylene Blue and 24.6% in Crystal Violet containing liquid media. In case of Crystal Violet, IUM1114 and IUM 2813 were found to have the same Crystal Violet degrading ability in solid media ++, whereas liquid medium showed two times better dye degradability in IUM 1114 strain. Congo Red decolorization ability was not found in the solid medium of IUM 1870, but was 30% in liquid dye culture. Therefore, the exact degree of decolorization could be distinguished through the culture of the liquid medium. However, although the same method was applied on media containing Remazol Brilliant Blue R dye, there was no difference in absorbance of the dye in media with or without fungus. It seems like these fungus strains did not decolorize this dye in the liquid media.

### Comparison of Phylogenetic Relation and Dye-Decolorization Ability in CCM Population

**Fig. 6:**
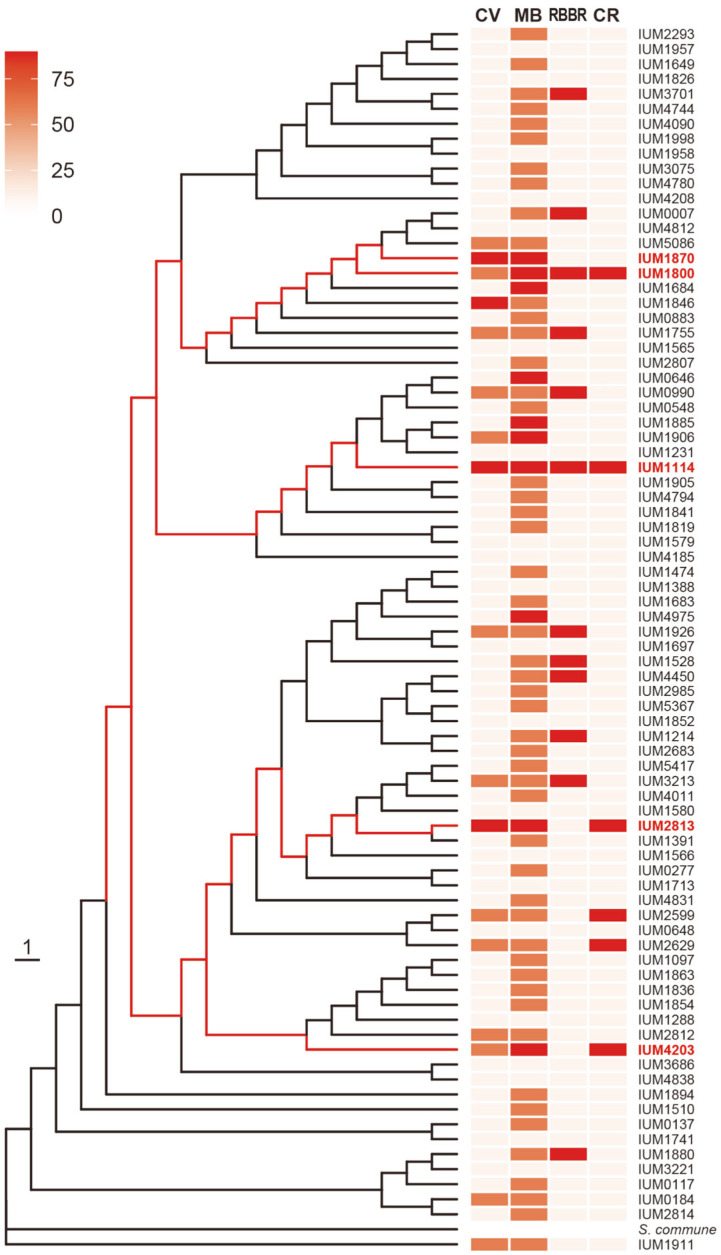
Dye-decolorization spectrum of 81 *S. commune* strains. Maximum likelihood tree was generated by RAxML using the CAT model and automatic bootstrapping options. In the heatmap, strong intensity indicates strong decolorizing ability. Five selected strains are colored in red. CR: Congo Red, RBBR: Remazol Brilliant Blue R, MB: Methylene Blue, CV: Crystal Violet.

In order to investigate whether dye decolorization ability correlates with the phylogenetic identification, the decolorization spectrum of 81 *S. commune* strains was compared with their ITS sequence based on the phylogenetic tree (Fig. 6). As shown in Fig. 6 and Supplemental Fig. 1, 81 strains isolated from CCM bank together formed several clades in the phylogenetic tree (Fig. 6), or haplotypes (Fig. S1). The decolorization spectrum of *S. commune* with 75% of strains showed the dye decolorization for least at one type of dye, presents a very diverse physiology phenotype of this population. However, the distribution of these strains along the tree is random. Especially, the 5 excellent strains showing strong ability of dye decolorization (Fig. 5) were separated from each other by different haplotype (or clade) as shown in red lines on the phylogenetic tree (Fig. 6). In addition, the 21 strains with no decolorization ability were distributed evenly. Therefore, there is no correlation between dye decolorization ability and ITS sequence based on the phylogenetic tree, suggesting that this ability was achieved differently and separately among strains during their evolution.

## Discussion

In this study, the use of a large population of *S. commune* enabled us to gain a comprehensive understanding of dye decolorization. Screening of dye decolorization for the 81 *S. commune* strains revealed their physiological diversity with 75% of the tested strains showing decolorization ability in at least one dye. Their decolorizing activities varied according to chromophore type of dyes, culture conditions, and strains. Phylogenetic tree and haplotype network analysis showed that those strains were genetically diverse and had genetic uniqueness in comparison with foreign population. Among them, we finally chose five excellent strains which showed the most powerful dye decolorization ability.

To study the DNA sequence variation at the intraspecific level, the combination of two types of graph phylogenetic trees and haplotype networks is useful for investigating the genetic diversity [21]. In this study, the phylogenetic tree showed the distance among strains (Figs. 1 and 3). Meanwhile, the haplotype network showed additional quantitative information such as: size of the population and the regional distribution of these *S. commune* strains (Fig. 2 and Table 1). Our strategy for genetic diversity was to compare the diversity of sub-populations with that of the whole (mixed) population. By doing these complicated comparisons, we could evaluate how much each population contributed to the whole population in terms of diversity. Table 1 shows how the diversity of the *S. commune* population in one country compares to that of the global population. In the number of haplotypes, the sum of domestic and foreign population (117) exceeds the number of the mixed population by three, indicating a very minor overlap between domestic and foreign populations. Five haplotypes (IX, LXXVIII, LXI, LXXXVI, and XCV) were shared by the domestic and foreign populations. This is similar with the results discovered by Alam *et al*. (2010) who showed that most of ten Korean and two Chinese *S. commune* strains formed a separate clade from the control strains (some of which were South American strains). This few-overlapped-phenomenon suggested that although the domestic population shows less nucleotide diversity than the foreign population, the domestic population had its uniqueness in ITS sequences, and therefore strongly contributed to the diversity of haplotype overall. Additionally, we could find the same phenomenon in Indian strains in the foreign population. Some 22 haplotypes were observed in the Indian population and 17 of them are unique. Both Korean and Indian populations have 75 and 34 strains, respectively. Judging from this, the more individuals involved, the more diverse the haplotypes that appear in *S. commune* population. Haplotype diversity, which was almost the same as ~0.92, supports the finding that the total population is highly diverse. Therefore, the *S. commune* population is highly diverse and the local population therefore has a higher chance of having unique haplotypes.

We also found that ITS is still a good barcode for molecular phylogenetic analysis in *S. commune* population. In fungi, the ribosomal RNA coding cistron is widely used for molecular systematic studies as well as population genetics. ITS is a very variable region that exists among rDNA sequences. Although ITS was stated to be less variable than the IGS1 region in the previous study (James *et al*., 2000), it was still very useful for assessing phylogenetic relationship at the intraspecific level [22s, 23-26]. When comparing the phylogenetic tree based on ITS sequences and regional distribution of the total 217 strains (Fig. 3), it also clearly showed several clades of which most strains are distributed in certain regions such as Europe and East Asia, South Asia and America. Therefore, using ITS sequence as a barcode, the genetic diversity of total strains can still be detected, and it helps to classify the genetic distribution of strains in Europe and Asia, which are normally classified as unclear when using IGS1 sequence [10]. This unclear distribution under IGS1 barcode can be used to explain the complication of phylogenetic tree in EAS (Europe/Asia/Australia) group. Maybe, the less variation of ITS compared with IGS1 simplifies analyzing the strains from EAS group. The use of ITS led us to group all American samples into one clade, instead of two big clades, NAM and SAM, as shown in James *et al*. (2001).

Although *S. commune* belonged to the WRF group and might strongly express the lignocellulose degrading ability, it has not been so much attractive in terms of dye decolorization ability. Up until now, only five *S. commune* strains have been the focus of study on dye decolorization ability, and these are IBL-06, F17, DMRF-7, CCB307, and one strain isolated from India (unknown name). IBL-06 strain from Pakistan, with optimized condition for enzyme activity showed dye degradation ability on several textile dyes such as: solar brilliant red 80, Cibacron Red, and Sandal-fix Foron blue [13, 14, 27]. F17 strain from China, with MnP playing a crucial role in decolorization of azo dyes, showed dye decolorization of Congo Red, Alizarin Red, Neutral Red, Orange G, Orange IV, and Crystal Violet. In particular, 80% of Congo Red was degraded under optimized condition [15, 16]. DMRF-7 strain from India showed high decolorization and degradation ability on two types of dyes, Rhodamine B and Methyl Violet, which was confirmed via Fourier transform infrared spectroscopy [18]. The unknown name of *S. commune* strain isolated from India, which is more efficient than another WRF *Lenzites* eximia in dye treatment, showed decolorization of several dyes including Congo Red, Methyl Orange, and Enrichrome Black-T via both adsorption of the dyes to the mycelial surface and metabolic breakdown [17]. These studies are sparse, random, and completely dependent on the luck of which strains are obtained. Only CCB307 strain from Brazil was discovered to have ability of decolorization on Remazol Brilliant Blue R when a list of basidiomycetes was screened [28]. But except this dye, there is no information of screening on other dyes. Therefore, screening of dye decolorization of *S. commune* strains is very necessary to provide a biotechnology resource for bioremediation.

In our study, we examined 75 Korean and 6 foreign strains of *S. commune* for four dyes, Congo Red, Remazol Brilliant Blue R, Methylene Blue and Crystal Violet. Our screening test showed an abundance of strains (75%examined strains) that had dye decolorization ability (Figs. 4B and 6). The dye decolorization rate is different according to strains, chromophores of dyes, and culture conditions. In the same media condition, different strains expressed different responses to dye decolorization. For example, IUM1870 strongly decolorized Methylene Blue while IUM1840 did not show any response to this dye on the same solid media (Fig. 4A). Chromophores in dye, the core part of a molecule responsible for its color, also affect decolorization ability of strains. Among four dyes, Methylene Blue containing Thiazine chromophore was considered as the easiest chromophore type to be decolorized by *S. commune* while Congo Red containing diazo is the most difficult (Fig. 4B). The dye decolorization ability of all examined strains has been displayed as spectrum, as shown in Fig 6, exposed the physiological diversity of the *S. commune* population. The dye decolorization ability of each strain also depended on culture condition, and when optimized helps enzymes perform their best. The different response of each tested strain on solid and liquid media implies that the culture condition is very important for strains to express their genes (Fig. 5). For example, IUM1114 and IUM 2813 were found to have the same Crystal Violet degrading ability in solid media ++, whereas liquid medium showed two times better dye degradability in IUM 1114 strain; decolorization of Congo Red was not found in the solid medium of IUM 1870, but was 30% in liquid culture. Remazol Brilliant Blue R was thought to be decolorized by IUM1114 and IUM1800 on solid media, but not in liquid. However, Remazol Brilliant Blue R was confirmed to be degraded by synthetic xylanase encoding xynA gene isolated from *S. commune* and expressed in E. coli [29]. Machado *et al*. (2005) reported that one strain of *S. commune* CCB307 could decolorize this dye while another CCB473 was not able decolorize in the same solid media, although no information about growing on liquid media was provided [28]. Excepting these two studies, and our screening (Figs. 4 and 5A), there is no other research mentioning the Remazol Brilliant Blue R decolorization or degradation ability of *S. commune* strains. The reason why IUM1114 and IUM1800 were incapable in Remazol Brilliant Blue R containing liquid media needs further elucidation. The benefit of growing on solid media is the quick observation result, which is very suitable for screening with a big amount of sample and monitoring decolorization during culture. The liquid medium not only helps to avoid some disadvantages of solid medium such as uneven distribution of fungal cells, transparency of media and impossibility of quantitative dye decolorization, but it also gets rid of debris and therefore provides a precise absorbance measurement result.

Through this study, we presented that the Korean white-rot mushroom has genetic diversity and a wide spectrum of decolorization for different dyes. The finding of dye decolorization ability in more than 60 strains is impressive. There was no clear correlation between the spectrum and the ITS-based phylogenetic tree (Fig. 6). This phenotypic diversity might be explained by genotypic variation of some oxidase genes. Such diverse phenotypic spectrum implied that diverse genotypes would be good resources for further study to explore genomes of *S. commune* population. Comparison of different strains might lead us to understand the mechanism of dye degradation and to open the possibility of genetic resources for industrial use in dye degradation as well as bioremediation.

## Supplemental Materials



Supplementary data for this paper are available on-line only at http://jmb.or.kr.

## Figures and Tables

**Table 1:** Comparison of genetic diversity among domestic and foreign *S. commune* strains.

Populations	Assigned strains	Length (nucleotide)	Nucleotide diversity	Haplotype diversity	

			Mean	Number	Diversity
Domestic population	75	503	0.000893	44	0.92505
Foreign population	138	505	0.002658	73	0.92066
Mixed population	217	501	0.002831	113	0.91867
