# Supplementary material for: Genetic Diversity and Dye-Decolorizing Spectrum of Schizophyllum commune Population
Source: J Microbiol Biotechnol. 2020 Aug 14;30(10):1525–35. doi: 10.4014/jmb.2006.06049 (PMC9728380; doi:10.4014/jmb.2006.06049)
Supplement: Supplementary file 1 [file JMB-30-10-1525-supple.pdf]

J. Microbiol. Biotechnol.
https://doi.org/10.4014/jmb.2006.06049

Supplementary Table 1: List of *Schizophyllum commune* isolated from culture collection of mushroom

Number	ID	Region Name	Haplotype ID in domestic population	Haplotype ID in Mixed population	Accession No
1	IUM2599	Gyeongsang	$I	$XXII	MK910820
2	IUM1911	Gyeongin	$II	$VI	MK910814
3	IUM1819	Gyeongin	$III	$XXIV	MK910800
4	IUM1579	Gyeongin	$IV	$XXX	MK910790
5	IUM4812	Gyeongin	$V	$XIV	MK910842
6	IUM4185	Gyeongin	$VI	$XXXII	MK910835
7	IUM2629	Gyeongin	$VII	$XXXV	MK910821
8	IUM1998	Jeolla	$VIII	$VII	MK910818
9	IUM1894	North Korea	$IX	$XXXVI	MK910811
10	IUM1565	Gangwon	$X	$XXXIX	MK910788
11	IUM1510	Gyeongin	$XI	$XXXVIII	MK910786
12	IUM1958	Gyeongsang	$XII	$VIII	MK910817
13	IUM4838	Gyeongin	$XIII	$IX	MK910844
14	IUM4090	Gyeongin	$XIV	$X	MK910834
15	IUM1649	Chungcheong	$XV	$XI	MK910792
16	IUM3701	North Korea	$XV	$XI	MK910832
17	IUM4744	Gyeongin	$XV	$XI	MK910839
18	IUM2293	Gyeongin	$XV	$XII	MK910819
19	IUM1957	Jeolla	$XV	$XII	MK910816
20	IUM1826	Chungcheong	$XVI	$XIII	MK910801
21	IUM0648	Gyeongin	$XVII	$XIX	MK910776
22	IUM0007	Jeju	$XVIII	$XVIII	MK910769
23	IUM3686	North Korea	$XIX	$XLII	MK910831
24	IUM2814	Gyeongsang	$XX	$LXXVIII	MK910826
25	IUM0184	Jeju	$XX	$LXXVIII	MK910772
26	IUM0117	Gyeongin	$XX	$LXXVIII	MK910770
27	IUM3221	Gyeongsang	$XX	$LXXVIII	MK910830
28	IUM1880	Chungcheong	$XXI	$LXXX	MK910809
29	IUM0990	Jeolla	$XXII	$LXI	MK910778
30	IUM1885	Chungcheong	$XXII	$LXI	MK910810
31	IUM0548	Gyeongin	$XXII	$LXI	MK910774
32	IUM0646	Gyeongin	$XXII	$LXI	MK910775
33	IUM1906	Gyeongsang	$XXIII	$LXIV	MK910813
34	IUM4831	Gyeongin	$XXIV	$LXV	MK910843
35	IUM4794	Gyeongin	$XXII	$LXI	MK910841
36	IUM1905	Chungcheong	$XXII	$LXI	MK910812
37	IUM1863	Jeolla	$XXV	$LXVI	MK910807
38	IUM1841	Chungcheong	$XXVI	$LXVII	MK910803
39	IUM1114	Gyeongin	$XXII	$LXI	MK910780
40	IUM1097	Jeolla	$XXVII	$LXVIII	MK910779
41	IUM0883	Gyeongin	$XXVIII	$LVIII	MK910777
42	IUM0137	Gyeongin	$XXIX	$C	MK910771
43	IUM0277	Gyeongin	$XXX	$LXXXVI	MK910773
44	IUM1214	Jeolla	$XXX	$LXXXVI	MK910781
45	IUM1683	Chungcheong	$XXX	$LXXXVI	MK910793
46	IUM2807	Chungcheong	$XXXI	$XCIV	MK910823
47	IUM2812	Gyeongsang	$XXXII	$CI	MK910824
48	IUM2985	Gyeongin	$XXX	$LXXXVI	MK910827
49	IUM3075	Gyeongin	$XXXIII	$XCV	MK910828
50	IUM4011	Gyeongin	$XXX	$LXXXVI	MK910833
51	IUM5417	Gyeongin	$XXX	$LXXXVI	MK910848
52	IUM5367	Gyeongin	$XXX	$LXXXVI	MK910847
53	IUM5086	Gyeongin	$XXXIV	$LXXXVIII	MK910846
54	IUM4975	Jeolla	$XXX	$LXXXVI	MK910845

55	IUM4450	Jeolla	$XXX	$LXXXVI	MK910838
56	IUM3213	Gyeongin	$XXX	$LXXXVI	MK910829
57	IUM2813	Chungcheong	$XXXV	$LXXXVI	MK910825
58	IUM2683	Gyeongsang	$XXXVI	$XCIII	MK910822
59	IUM1870	Chungcheong	$XXXVII	$LXXXII	MK910808
60	IUM1854	Gyeongin	$XXXVIII	$XCII	MK910806
61	IUM1852	Gyeongin	$XXX	$LXXXVI	MK910805
62	IUM1846	Chungcheong	$XXXIX	$LXXXI	MK910804
63	IUM1836	Chungcheong	$XL	$LXXXVII	MK910802
64	IUM1755	Chungcheong	$XLI	$CXII	MK910798
65	IUM1713	Jeolla	$XXX	$LXXXVI	MK910796
66	IUM1697	Gyeongsang	$XXX	$LXXXVI	MK910795
67	IUM1580	Gyeongin	$XXX	$LXXXVI	MK910791
68	IUM1566	Gangwon	$XXX	$LXXXVI	MK910789
69	IUM1528	Gyeongin	$XXX	$LXXXVI	MK910787
70	IUM1474	Gyeongin	$XXX	$LXXXVI	MK910785
71	IUM1391	Gyeongin	$XXX	$LXXXVI	MK910784
72	IUM1388	Gyeongin	$XXX	$LXXXVI	MK910783
73	IUM1288	Chungcheong	$XLII	$LXXXV	MK994018
74	IUM1684	Jeolla	$XLIII	$LXXXIII	MK910794
75	IUM1800	Jeolla	$XLIV	$LXXXIV	MK910799
76	IUM1231	Thailand	NA	$LXI	MK910782
77	IUM1741	Myanmar	NA	$XLIX	MK910797
78	IUM1926	China	NA	$LXXXVI	MK910815
79	IUM4203	Italy	NA	$XLVIII	MK910836
80	IUM4208	Taiwan	NA	$XXXI	MK910837
81	IUM4780	Vietnam	NA	$XCV	MK910840

NA: not applied

Supplementary Table 2. List of *Schizophyllum commune* isolates download from NCBI

Number	ID	Regions	Haplotype ID in foreign population	Haplotype ID in Mixed population
1	KP172539.1	East Asia	$I	$I
2	KC414807.1	South Asia	$II	$II
3	JF766994.1	America	$III	$III
4	MF098693.1	Europe	$IV	$XXI
5	EU520221.1	East Asia	$V	$XXXIV
6	KC414803.1	South Asia	$VI	$IV
7	JQ341139.1	Africa	$VII	$XXVI
8	AF249374.1	Europe	$VIII	$XXVII
9	AF249384.1	America	$IX	$V
10	AF249388.1	America	$X	$XXIII
11	KC414805.1	South Asia	$XI	$XXV
12	IUM4208	East Asia	$XII	$XXXI
13	AF249366.1	East Asia	$XIII	$XXVIII
14	AF249385.1	America	$XIV	$XXIX
15	AF249386.1	America	$XIV	$XXIX
16	KX028769.1	Europe	$XV	$IX
17	KX028770.1	Europe	$XV	$IX
18	KX668572.1	America	$XVI	$LVI
19	AF249368.1	Oceania_Antar	$XVII	$XXXIII
20	AF249379.1	America	$XVIII	$XVII
21	KC414796.1	South Asia	$XIX	$CIV
22	KC414798.1	South Asia	$XX	$XXXVII
23	KC414799.1	South Asia	$XXI	$LIII
24	KC414794.1	South Asia	$XXII	$LVII
25	KC414795.1	South Asia	$XXII	$LVII
26	MF037414.1	Africa	$XXIII	$CX
27	AF249372.1	Oceania_Antar	$XXIV	$XL
28	AF249387.1	America	$XXV	$LII
29	KC414812.1	South Asia	$XXVI	$XLI
30	KC414792.1	South Asia	$XXVII	$CII
31	MF185106.1	South Asia	$XXVIII	$XCV
32	KY352530.1	Europe	$XXIX	$LV
33	KX555523.1	Africa	$XXX	$XLIII
34	KX555521.1	Africa	$XXXI	$LXIII
35	KX555524.1	Africa	$XXXII	$XLIV
36	KX555522.1	Africa	$XXIX	$LV
37	KM985684.1	South Asia	$XXXIII	$LIV
38	MH507017.1	South Asia	$XXXIV	$LIX
39	IUM4203	Europe	$XXXV	$XLVIII
40	AF249390.1	America	$XXXVI	$XLVII
41	IUM1741	South Asia	$XXXVII	$XLIX
42	KX668573.1	America	$XXXVIII	$L
43	MH325921.1	Europe	$XXXIX	$LI
44	KT385794.1	South Asia	$XL	$CVII
45	KT385796.1	South Asia	$XL	$CVII
46	KT385802.1	South Asia	$XLI	$CVIII
47	MH142024.1	East Asia	$XLII	$LXXVIII
48	AF249365.1	East Asia	$XLII	$LXXVIII
49	AF249367.1	South Asia	$XLII	$LXXVIII

50	AF249373.1	Africa	$XLII	$LXXVIII
51	AF249375.1	South Asia	$XLII	$LXXVIII
52	MF920418.1	Oceania_Antar	$XLII	$LXXVIII
53	KC414800.1	South Asia	$XLIII	$LXXIX
54	KU844329.1	South Asia	$XLII	$LXXVIII
55	KC414802.1	South Asia	$XLII	$LXXVIII
56	KC414813.1	South Asia	$XLII	$LXXVIII
57	MF773657.1	South Asia	$XLIV	$LXXVIII
58	KC414797.1	South Asia	$XLV	$XLV
59	KC414809.1	South Asia	$XLV	$XLV
60	AF249380.1	America	$XLVI	$XV
61	AF249382.1	America	$XLVII	$XVI
62	AF249381.1	America	$XLVIII	$XX
63	KC414793.1	South Asia	$XLIX	$CIII
64	KC414806.1	South Asia	$L	$CV
65	KC414811.1	South Asia	$LI	$CXI
66	KP036942.1	South Asia	$LII	$CXIII
67	KT343971.1	South Asia	$XXVIII	$XCV
68	MH221094.1	South Asia	$XXVIII	$XCV
69	KC414810.1	South Asia	$XXVIII	$XCV
70	KM985683.1	South Asia	$XXVIII	$XCV
71	MF773673.1	South Asia	$XXVIII	$XCV
72	KT385772.1	South Asia	$XXVIII	$XCV
73	KC414814.1	South Asia	$XXVIII	$XCV
74	IUM4780	South Asia	$XXVIII	$XCV
75	KY425733.1	South Asia	$XXVIII	$XCV
76	KT385793.1	South Asia	$XXVIII	$XCV
77	KF291014.1	South Asia	$XXVIII	$XCV
78	KC414808.1	South Asia	$XXVIII	$XCV
79	KJ865831.1	South Asia	$XXVIII	$XCV
80	AF249389.1	America	$LIII	$XLVI
81	KX668571.1	America	$LIV	$LXXIV
82	MF423713.1	Europe	$LV	$LXXXVI
83	EU520209.1	East Asia	$LVI	$XCIX
84	AF249364.1	East Asia	$LVII	$XCI
85	MF098694.1	Europe	$LVIII	$XCVI
86	EU520217.1	East Asia	$LV	$LXXXVI
87	FJ462753.1	East Asia	$LV	$LXXXVI
88	KX258807.1	East Asia	$LV	$LXXXVI
89	EU520239.1	East Asia	$LIX	$LX
90	AF249369.1	Oceania_Antar	$LX	$XCVIII
91	IUM1231	South Asia	$LXI	$LXI
92	KX028779.1	Europe	$LXII	$CIX
93	KX028780.1	Europe	$LXII	$CIX
94	KX028781.1	Europe	$LXII	$CIX
95	GU062310.1	Europe	$LXIII	$LXX
96	KM985685.1	South Asia	$LXIV	$LXXIII
97	MF773651.1	South Asia	$LXV	$XCVII
98	AF249377.1	America	$LV	$LXXXVI
99	AF249376.1	Europe	$LV	$LXXXVI
100	AF249371.1	Oceania_Antar	$LV	$LXXXVI
101	AF249370.1	Oceania_Antar	$LV	$LXXXVI
102	EU520253.1	East Asia	$LV	$LXXXVI

103	KC414801.1	South Asia	$LXVI	$LXXXIX
104	KC414804.1	South Asia	$LV	$LXXXVI
105	KF513165.1	East Asia	$LV	$LXXXVI
106	KT323147.1	Europe	$LV	$LXXXVI
107	KT323207.2	Europe	$LV	$LXXXVI
108	KX028768.1	Europe	$LV	$LXXXVI
109	KX028771.1	Europe	$LV	$LXXXVI
110	KX028772.1	Europe	$LV	$LXXXVI
111	KX028773.1	Europe	$LV	$LXXXVI
112	KX028774.1	Europe	$LV	$LXXXVI
113	KX028775.1	Europe	$LV	$LXXXVI
114	KX028776.1	Europe	$LV	$LXXXVI
115	KX028777.1	Europe	$LV	$LXXXVI
116	KX028778.1	Europe	$LV	$LXXXVI
117	KX555525.1	Africa	$LV	$LXXXVI
118	KY381868.1	East Asia	$LV	$LXXXVI
119	MF098692.1	Europe	$LV	$LXXXVI
120	MF423708.1	Europe	$LV	$LXXXVI
121	MF423709.1	Europe	$LV	$LXXXVI
122	MF423711.1	Europe	$LV	$LXXXVI
123	MF423712.1	Europe	$LV	$LXXXVI
124	MF423714.1	Europe	$LV	$LXXXVI
125	MF423715.1	Europe	$LV	$LXXXVI
126	MF423716.1	Europe	$LV	$LXXXVI
127	JF440579.1	Europe	$LV	$LXXXVI
128	KR296883.1	South Asia	$XLII	$LXXXVI
129	IUM1926	East Asia	$XLII	$LXXXVI
130	KY864394.1	South Asia	$LXVII	$LXXV
131	AF249383.1	America	$LXVIII	$LXIX
132	KJ832063.1	America	$LXIX	$LXXII
133	KJ417836.1	Oceania_Antar	$LXX	$LXXI
134	MF347412.1	America	$LXXI	$LXXVII
135	S.commune	no region	$LXXII	$LXXVI
136	MF423707.1	Europe	$LXXIII	$XC
137	AF249378.1	America	$LV	$LXXXVI
138	MF423710.1	Europe	$LV	$LXXXVI
139	KR673670.1	East Asia (South Korea)	NA	$CVI
140	KR673682.1	East Asia (South Korea)	NA	$LXII
141	KJ714011.1	East Asia (South Korea)	NA	$LXXVIII
142	KP004975.1	East Asia (South Korea)	NA	$LXXXVI

NA: not applied

Supplementary Table 3. Geographic information of *Schizophyllum commune* population from NCBI

Continents	Countries	No. of isolates
Africa (N = 8)	Cameroon	1
	Ghana	1
	Nigeria	1
	Tunisia	5
East Asia (N = 18)	China	13
	Japan	1
	**South Korea**	**4**
South Asia (N = 46)	India	34
	Malaysia	1
	Singapore	1
	Sri Lanka	4
	Taiwan	1
	Thailand	5
Europe (N = 36)	Cyprus	1
	Czech Republic	1
	France	2
	Germany	1
	Italy	11
	Latvia	1
	Lithuania	1
	England	1
	Poland	14
	Spain	2
	Sweden	1
America (N = 22)	Antarctica	2
	Argentina	2
	Bahamas	1
	Belize	1
	Brazil	6
	Chile	1
	Costa Rica	1
	Puerto Rico	2
	USA	6
Oceania (N = 5)	Australia	1
	New Zealand	3
	Papua New Guinea	1
	Total	135

Supplementary figure 1

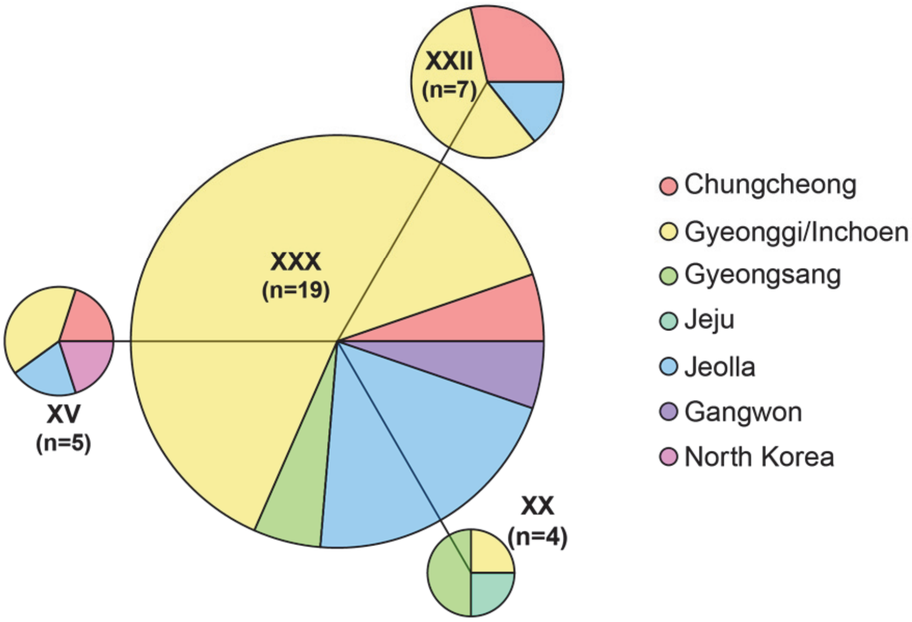

Fig S1. Haplotype network analysis of domestic white-rot fungus. Total of 44 haplotypes distributed evenly. Only haplotypes containing more than 2 strains were displayed on the graph.

Supplementary figure 2

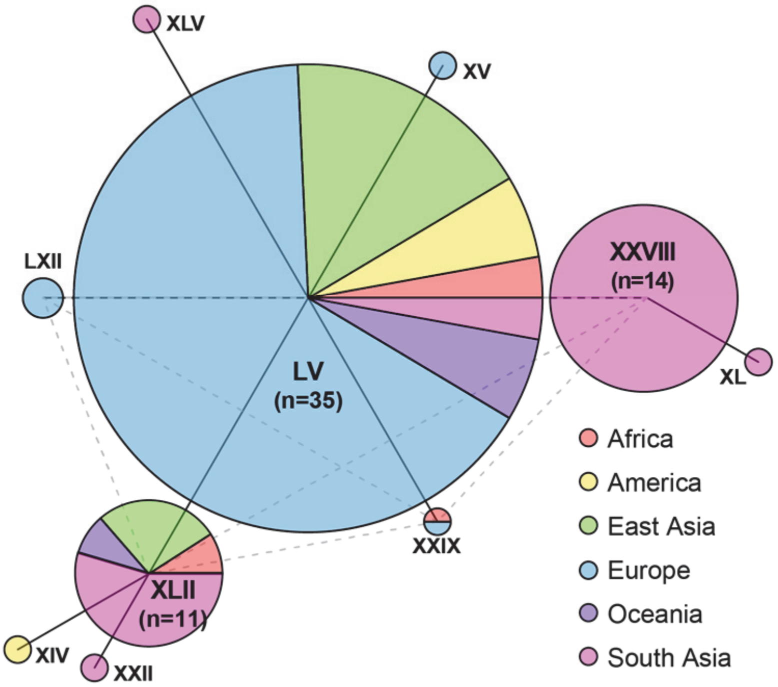

Fig S2. Haplotype network analysis of 138 foreign white-rot fungus strains. Number of haplotypes is 73. Only haplotypes containing more than 2 strains were displayed on the graph.
